# Making sense of big data in health research: Towards an EU action plan

**DOI:** 10.1186/s13073-016-0323-y

**Published:** 2016-06-23

**Authors:** Charles Auffray, Rudi Balling, Inês Barroso, László Bencze, Mikael Benson, Jay Bergeron, Enrique Bernal-Delgado, Niklas Blomberg, Christoph Bock, Ana Conesa, Susanna Del Signore, Christophe Delogne, Peter Devilee, Alberto Di Meglio, Marinus Eijkemans, Paul Flicek, Norbert Graf, Vera Grimm, Henk-Jan Guchelaar, Yi-Ke Guo, Ivo Glynne Gut, Allan Hanbury, Shahid Hanif, Ralf-Dieter Hilgers, Ángel Honrado, D. Rod Hose, Jeanine Houwing-Duistermaat, Tim Hubbard, Sophie Helen Janacek, Haralampos Karanikas, Tim Kievits, Manfred Kohler, Andreas Kremer, Jerry Lanfear, Thomas Lengauer, Edith Maes, Theo Meert, Werner Müller, Dörthe Nickel, Peter Oledzki, Bertrand Pedersen, Milan Petkovic, Konstantinos Pliakos, Magnus Rattray, Josep Redón i Màs, Reinhard Schneider, Thierry Sengstag, Xavier Serra-Picamal, Wouter Spek, Lea A. I. Vaas, Okker van Batenburg, Marc Vandelaer, Peter Varnai, Pablo Villoslada, Juan Antonio Vizcaíno, John Peter Mary Wubbe, Gianluigi Zanetti

**Affiliations:** European Institute for Systems Biology and Medicine, 1 avenue Claude Vellefaux, 75010 Paris, France; CIRI-UMR5308, CNRS-ENS-INSERM-UCBL, Université de Lyon, 50 avenue Tony Garnier, 69007 Lyon, France; Luxembourg Centre for Systems Biomedicine, University of Luxembourg, 7 Avenue des Hauts Fourneaux, 4362 Esch-sur-Alzette, Luxembourg; Wellcome Trust Sanger Institute, Wellcome Genome Campus, Hinxton, Cambridge, CB10 1SA UK; Health Services Management Training Centre, Faculty of Health and Public Services, Semmelweis University, Kútvölgyi út 2, 1125 Budapest, Hungary; Centre for Personalised Medicine, Linköping University, 581 85 Linköping, Sweden; Translational & Bioinformatics, Pfizer Inc., 300 Technology Square, Cambridge, MA 02139 USA; Institute for Health Sciences, IACS - IIS Aragon, San Juan Bosco 13, 50009 Zaragoza, Spain; ELIXIR, Wellcome Genome Campus, Hinxton, Cambridge, CB10 1SD UK; CeMM Research Center for Molecular Medicine of the Austrian Academy of Sciences, Lazarettgasse 14, AKH BT25.2, 1090 Vienna, Austria; Department of Laboratory Medicine, Medical University of Vienna, Lazarettgasse 14, AKH BT25.2, 1090 Vienna, Austria; Max Planck Institute for Informatics, Campus E1 4, 66123 Saarbrücken, Germany; Príncipe Felipe Research Center, C/ Eduardo Primo Yúfera 3, 46012 Valencia, Spain; University of Florida, Institute of Food and Agricultural Sciences (IFAS), 2033 Mowry Road, Gainesville, FL 32610 USA; Bluecompanion Ltd, 6 London Street (second floor), London, W2 1HR UK; Technology, Data & Analytics, KPMG Luxembourg, Société Coopérative, 39 Avenue John F. Kennedy, 1855 Luxembourg, Luxembourg; Department of Human Genetics, Department of Pathology, Leiden University Medical Centre, Einthovenweg 20, 2333 ZC Leiden, The Netherlands; Information Technology Department, European Organization for Nuclear Research (CERN), 385 Route de Meyrin, 1211 Geneva 23, Switzerland; Julius Center for Health Sciences and Primary Care, University Medical Center Utrecht, Heidelberglaan 100, 3508 GA Utrecht, The Netherlands; European Molecular Biology Laboratory, European Bioinformatics Institute (EMBL-EBI), Wellcome Genome Campus, Hinxton, Cambridge, CB10 1SD UK; Department of Pediatric Oncology/Hematology, Saarland University, Campus Homburg, Building 9, 66421 Homburg, Germany; Project Management Jülich, Forschungszentrum Jülich GmbH, Wilhelm-Johnen-Straße, 52428 Jülich, Germany; Department of Clinical Pharmacy & Toxicology, Leiden University Medical Center, Albinusdreef 2, 2333 ZA Leiden, The Netherlands; Data Science Institute, Imperial College London, South Kensington, London, SW7 2AZ UK; CNAG-CRG, Center for Genomic Regulation, Barcelona Institute for Science and Technology (BIST), C/Baldiri Reixac 4, 08029 Barcelona, Spain; Institute of Software Technology and Interactive Systems, TU Wien, Favoritenstrasse 9-11/188, 1040 Vienna, Austria; The Association of the British Pharmaceutical Industry, 7th Floor, Southside, 105 Victoria Street, London, SW1E 6QT UK; Department of Medical Statistics, RWTH-Aachen University, Universitätsklinikum Aachen, Pauwelsstraße 30, 52074 Aachen, Germany; SYNAPSE Research Management Partners, Diputació 237, Àtic 3ª, 08007 Barcelona, Spain; Department of Infection, Immunity and Cardiovascular Disease and Insigneo Institute for In-Silico Medicine, Medical School, University of Sheffield, Beech Hill Road, Sheffield, S10 2RX UK; Department of Statistics, School of Mathematics, University of Leeds, Leeds, LS2 9JT UK; Department of Medical & Molecular Genetics, King’s College London, London, SE1 9RT UK; Genomics England, London, EC1M 6BQ UK; National and Kapodistrian University of Athens, Medical School, Xristou Lada 6, 10561 Athens, Greece; Vitromics Healthcare Holding B.V., Onderwijsboulevard 225, 5223 DE ’s-Hertogenbosch, The Netherlands; Fraunhofer Institute for Molecular Biology and Applied Ecology ScreeningPort, Schnackenburgallee 114, 22525 Hamburg, Germany; ITTM S.A., 9 avenue des Hauts Fourneaux, 4362 Esch-sur-Alzette, Luxembourg; Research Business Technology, Pfizer Ltd, GP4 Building, Granta Park, Cambridge, CB21 6GP UK; Health Economics & Outcomes Research, Deloitte Belgium, Berkenlaan 8A, 1831 Diegem, Belgium; Janssen Pharmaceutica N.V., R&D G3O, Turnhoutseweg 30, 2340 Beerse, Belgium; Faculty of Life Sciences, University of Manchester, AV Hill Building, Oxford Road, Manchester, M13 9PT UK; UMR3664 IC/CNRS, Institut Curie, Section Recherche, Pavillon Pasteur, 26 rue d’Ulm, 75248 Paris cedex 05, France; Linguamatics Ltd, 324 Cambridge Science Park Milton Rd, Cambridge, CB4 0WG UK; PwC Luxembourg, 2 rue Gerhard Mercator, 2182 Luxembourg, Luxembourg; Philips, HighTechCampus 36, 5656AE Eindhoven, The Netherlands; Department of Public Health and Primary Care, KU Leuven Kulak, Etienne Sabbelaan 53, 8500 Kortrijk, Belgium; INCLIVA Health Research Institute, University of Valencia, CIBERobn ISCIII, Avenida Menéndez Pelayo 4 accesorio, 46010 Valencia, Spain; Swiss Institute of Bioinformatics (SIB) and University of Basel, Klingelbergstrasse 50/70, 4056 Basel, Switzerland; Agency for Health Quality and Assessment of Catalonia (AQuAS), Carrer de Roc Boronat 81-95, 08005 Barcelona, Spain; EuroBioForum Foundation, Chrysantstraat 10, 3135 HG Vlaardingen, The Netherlands; Integrated BioBank of Luxembourg, 6 rue Nicolas-Ernest Barblé, 1210 Luxembourg, Luxembourg; Technopolis Group, 3 Pavilion Buildings, Brighton, BN1 1EE UK; Hospital Clinic of Barcelona, Institute d’Investigacions Biomediques August Pi Sunyer (IDIBAPS), Rosello 149, 08036 Barcelona, Spain; European Platform for Patients’ Organisations, Science and Industry (Epposi), De Meeûs Square 38-40, 1000 Brussels, Belgium; CRS4, Ed.1 POLARIS, 09129 Pula, Italy; BBMRI-ERIC, Neue Stiftingtalstrasse 2/B/6, 8010 Graz, Austria

## Abstract

Medicine and healthcare are undergoing profound changes. Whole-genome sequencing and high-resolution imaging technologies are key drivers of this rapid and crucial transformation. Technological innovation combined with automation and miniaturization has triggered an explosion in data production that will soon reach exabyte proportions. How are we going to deal with this exponential increase in data production? The potential of “big data” for improving health is enormous but, at the same time, we face a wide range of challenges to overcome urgently. Europe is very proud of its cultural diversity; however, exploitation of the data made available through advances in genomic medicine, imaging, and a wide range of mobile health applications or connected devices is hampered by numerous historical, technical, legal, and political barriers. European health systems and databases are diverse and fragmented. There is a lack of harmonization of data formats, processing, analysis, and data transfer, which leads to incompatibilities and lost opportunities. Legal frameworks for data sharing are evolving. Clinicians, researchers, and citizens need improved methods, tools, and training to generate, analyze, and query data effectively. Addressing these barriers will contribute to creating the European Single Market for health, which will improve health and healthcare for all Europeans.

## European healthcare systems and the potential for big data

Medicine has traditionally been a science of observation and experience. For thousands of years, clinicians have integrated the knowledge of preceding generations with their own life-long experiences to treat patients according to the oath of Hippocrates; mostly based on trial and error. Knowledge generation is changing dramatically. The digitalization of medicine allows the comparison of disease progression or treatment responses from patients worldwide. Whole-genome sequencing allows searching and comparing one’s own genome to millions and soon billions of other human genomes. Eventually, the entire world population could be used as a reference population in order to link genome information with many other types of physiological, clinical, environmental, and lifestyle data. For many, this is a vision full of opportunities, whereas for others it provides a wealth of technical challenges, unanticipated consequences, and loss of privacy and autonomy.

The quality of conclusions on the etiology of diseases follows a law of large numbers. Cross-sectional cohort studies of 30,000 to 50,000 or more cases are required to separate the signal from noise and to detect genomic regions associated with a given trait in which disease-related genes or susceptibility factors are located [1, 2]. Whole-genome sequencing studies often identify only a few genomic regions that contain elements with large effects on the penetrance or expressivity of gene products but hundreds of genomic regions that have small effects and are highly dependent on genetic background, environmental factors, or social and lifestyle determinants [3]. There is also a need to study disease pathogenesis on genome, epigenome, transcriptome, proteome, and metabolome levels and combine these dimensions through multi-omics research. Furthermore, individual variation responsible for normal and disease phenotypes is high as a result of somatic mutations or variation in transcription, splicing, or allele-specific gene expression between individuals [4–6].

Vast amounts of temporal and spatial parameter data are now available. But what are we going to do with the data? It takes hard work to condense useful information from big data and turn this information into knowledge and action. The challenge will be to make a smart choice between situations when less is more versus less is less but also when more is more versus more is less.

Here, we briefly describe the key challenges that result from making sense out of big data in health and using these data for the benefit of the patient and the healthcare system. We also highlight key technical, legal, and ethical issues that we face to develop evidence-based personalized medicine. Finally, we put forward five recommendations for the European Union (EU) and member states’ policy makers to serve as a framework for an EU action plan that could help to reach this ambitious goal.

## Making sense of big data in health research

On 30 October 2015, the Health Directorate of the Directorate-General for Research and Innovation at the European Commission (EC), the executive body of the EU, organized in Luxembourg a workshop entitled “Big data in health research: an EU action plan” [7]. The aim of the workshop was to ask stakeholders in the “big data revolution” for their input on how European funding for health research should take into account the opportunities, limitations, and concerns of the anticipated developments in health and healthcare. Participants included bioinformaticians, computational biologists, genome scientists, drug developers, biobanking experts, experimental biologists, biostatisticians, information and communication technology (ICT) experts, public health researchers, clinicians, public policy experts, representatives of health services, patient advocacy groups, the pharmaceutical industry, and ICT companies.

## What do we mean by “big data”?

“Big data” has a wide range of definitions in health research [8, 9] and to create a single definition for all uses (“one size fits all” approach) may be too abstract to be useful. However, a workable definition of what big data means for health research or at least a consensus of what this term means was proposed during the workshop in Luxembourg. “Big data in health” encompasses high volume, high diversity biological, clinical, environmental, and lifestyle information collected from single individuals to large cohorts, in relation to their health and wellness status, at one or several time points. Big data can only be dealt with by adopting a strong governance model and best practices of new technologies, e.g., in large-scale data production compliant with community-based quality standards, coupled with interoperable data storage, data integration, and advanced analytics solutions [10]. Another goal of the workshop was to develop an EU action plan for research funders towards the integration of big data into policy development, biomedical research, and clinical practice in health and wellness management. Big data comes from a variety of sources, such as clinical trials, electronic health records (EHRs), patient registries and databases, multidimensional data from genomic, epigenomic, transcriptomic, proteomic, metabolomic, and microbiomic measurements, and medical imaging. More recently, data are being integrated from social media, socioeconomic or behavioral indicators, occupational information, mobile applications, or environmental monitoring [11]. Big data comes in a wide range of formats. Data streams have to be assessed and interpreted in a timely manner to benefit patients affected by diseases and to help citizens remain in good health [8, 12].

## Importance of patient registries

Patient registries have for decades served as a key tool for assessing clinical outcome and clinical and health technology performance [13–15]. Rare disease registries pool data to achieve a sufficient sample size for epidemiological and/or clinical research [16, 17]. The European Organization for Research and Treatment of Cancer (EORTC) [18] opened a prospective registry for patients with melanoma in June 2015 [19]. The European Network of Cancer Registries (ENCR) [20], established within the framework of the Europe Against Cancer Programme of the EC, promotes collaboration between cancer registries, defines data collection standards, provides training for cancer registry personnel, and regularly disseminates information on incidence and mortality from cancer in the European Union and other European countries.

Patient registries provide significant potential for research and public health improvements in the EU, owing to the large volume of patients in each registry and the variety of quality medical information related to each patient. Patient registries are increasingly important to monitor patients’ treatments and for safety assessment and the identification of trends in translational medicine (e.g., registry-based clinical trials, personalized medicine) [21].

Patient registries allow informed policy decisions at the local, regional, national, and, in some cases, the international level. As a result, hundreds of registries have been set up that range from national to international rare disease initiatives, coupling clinical and genetic data and biobanks. However, for various reasons, including data protection and the fragmentation of regulatory frameworks, the combination of these disparate information sources to guide health research and decision-making in the clinic has so far lagged behind the use of large-scale, big data collections in other sectors. Other disciplines, such as electronic and mechanical engineering, and whole industries, such as building airplanes, weather forecasting, or robotics, have demonstrated computational modeling and simulation as an essential component that is based on data sharing and their experience could help overcome the barriers experienced in health research [22–24].

## The potential benefits of big data for healthcare

Big data in health can be used to improve the efficiency and effectiveness of prediction and prevention strategies or of medical interventions, health services, and health policies [25–27]. Access to well-curated and high quality health-related data will likely have a number of benefits in a diverse range of situations. In clinical practice, these data will improve outcomes for individual patients through personalization of predictions, earlier diagnosis, better treatments, and improved decision support for clinicians in cyclic processes. (Cyclic processes are usually composed of the definition of policy/decision options, the selection of the best alternative, and the subsequent implementation and validation of this option. Integrating feedback from a continuous evaluation on the process completes this cycle [28]*.*) These improvements should eventually lead to lowered costs for the healthcare system.

Likewise, the integration of fragmented information systems into the clinical life cycle will allow the discovery of medically relevant associations, early signals, or changed disease trajectories and should, therefore, enable better patient management strategies and improved quality and safety of care. For clinical trials, more expansive, interoperable health records should make it much easier to find suitable participants and to design and assess the feasibility of new studies [29, 30]. Moreover, better management of big data would enable a more systematic identification of drug safety signals, such as earlier detection of adverse drug reactions [31], while allowing personalized medicine analyses via appropriate patient and/or population stratification methodologies. This in turn should lead to improved treatment responses for biologically or clinically defined patient subgroups, which will also avoid unnecessary rejection of potent drugs and devices. As a result, patient communities will benefit and the unsustainable trend of escalating costs in hospital and community care management as well as diagnostic and drug development costs by the biopharma industries will stop or slow down. Health economy specialists need to provide suitable metrics to monitor key performance indicators of success in big data pilot projects. Such metrics might include the change in response rates in stratified patient subpopulations or the number of adverse drug reactions after systems medicine-based companion diagnostics.

Big data also has many potential benefits for translational research into health and well-being. Integrated data sets should improve models of common disease to better understand the progression of rare diseases [32]. They may also enable the detection of population-level effects, such as the off-target and adverse effects of drugs or the occurrence of co-morbidity [33].

Biomarkers constitute a key building block of precision medicine, yet the development and clinical validation of new biomarkers is a lengthy process and relatively few such markers have yet reached routine clinical practice [34, 35]. However, a sizeable number of biomarkers are now widely used in routine clinical diagnostics, which include—but are not limited to—targeted cancer therapy [36–39]. Multidimensional signatures that take into account a wealth of prior information, both from the patient’s previous life history and state-of-the-art information from the literature and relevant databases, will hopefully deliver a much higher predictive power than the single biomarkers used today. There is also potential for research on the impact of healthcare interventions and monitoring trends in infectious diseases to inform public health policies [40].

Finally, there is an opportunity to engage with the individual patient more closely and import data from mobile health applications or connected devices. This interaction with the patient will result in the collection of more detailed clinical, environmental, and lifestyle information, such as heart frequency and body temperature, physical activity and nutrition habits, and sleep and stress management, which will prevent risk exposure and disease onset [41]. Personal monitoring over time should aid the early detection of deviations from a healthy state and trajectories should lead to actionable recommendations, making it possible for individuals to maintain themselves in good health [12].

## The challenges ahead for the effective use of big data in healthcare

To refine the recommendations for an EU action plan, we identified the main challenges that exist for the use of big data in healthcare research and in the clinic. The challenges have been reported elsewhere [42, 43] and include clinical, technical, legal, and cultural hurdles. These challenges vary depending on whether the data are preclinical based on cellular or animal models or from patients in clinical settings, on the intended type of analysis and interpretation, on cross-cultural aspects of privacy, and on ethical and legal considerations. We are on the cusp of having access to vast personal data—for example, on physiological, behavioral, molecular, clinical, environmental exposure, medical imaging, disease management, medication prescription history, nutrition, or exercise parameters—that could potentially be used to track the health of individuals and populations in considerably more detail than ever before. The integration of structured and unstructured data, using natural language processing and other sophisticated machine learning tools, is being tested and it is hoped this will lead to a new level of integration of prior information with up-to-date clinical information [44].

Over a thousand Mendelian disorders are linked to genetic defects and, for many of these, genetic testing is performed to inform clinical practice. The most successful integration of basic and clinical data can be observed in oncology [45, 46] and in research on rare diseases [47–49]. However, the medical relevance of the large amount of genetic variation revealed by genomic sequencing is still unknown in most cases.

Data acquisition is undergoing rapid change. Wearable devices, integrated sensors, and continuous monitoring capabilities are available for all scales of measurements [50]. Several legal issues will have to be tackled, for example, when a consumer device becomes a diagnostic device and the quality assurance and regulatory approval are more stringent [51].

Data storage issues include security, accessibility, and sustainability. Should data be stored centrally or in a federated manner? There are concerns about entrusting health-related data to public clouds. As a result, there is a strong need to come up with alternatives. The decades of experience in big data management for the particle physics community at The European Organization for Nuclear Research (CERN) that led to the development of the World Wide Web [52] will be valuable. However, many aspects that are specific to big data in health research need to be taken into account, such as data heterogeneity, institutional and legal fragmentation, and strong data protection standards. There will be a massive increase in big data production in all areas of biomedical research, which includes studies at the preclinical levels, such as animal or cellular models and translational studies, but also clinical research that involves patients or public health research. To make the most use of the information produced, several technical challenges should be addressed, such as the combination of structured data, such as genotype, phenotype, and genomics data, with semi-structured and unstructured data, e.g., medical imaging, EHRs, lifestyle, environmental, and health economics data [53–56]. Recent successful examples show the feasibility of combining such data for translational and clinical research [57–59].

## Technical challenges related to the management of electronic health records

Adoption of EHRs across Europe varies greatly. Estonia [60] and the Valencia Community in Spain (Josep Redón i Màs, personal communication) have moved entirely to EHRs. Integration is supported with auxiliary systems, for instance drug–drug interaction alert systems that warn physicians and pharmacists about potential prescription clashes, clinical risk groups calculation and costs (e.g., Valencia region, Spain), and drug–gene interaction alert systems that guide physicians to adjust the dose of a prescribed drug in aberrant drug metabolizers (e.g., The Netherlands). The USA have taken steps towards a “patient-driven economy” [61]. In such a scenario, the patient owns his/her data. This ownership requires the development of an appropriate health-record infrastructure but provides a wide range of new health service business opportunities with major economic potential. Empowering patients to take control of their data could be of particular importance for cross-border healthcare and health research activities in Europe where healthcare is highly fragmented and multinational. To transfer medical data from one country to another in the EU is very difficult. Ownership of data by patients could overcome these obstacles and unleash new ways to stimulate a competitive health-driven economy.

Furthermore, patient records can be computationally opaque, for example, in the form of free text, recorded speech, or medical images; translation into a format compatible with computational analyses will be necessary. Data in different languages and time-consuming searches and identification are other important barriers.

There are some best practices for the management of EHRs. For example, the International Rare Diseases Research Consortium (IRDiRC) [62] develops and implements standards and harmonized methodology across diseases and medical cases [63]. Several European collaborative projects, such as the European project p-medicine, have created IT infrastructures that will facilitate translational research and the development of personalized medicine [64]. ELIXIR, one of the European infrastructures for life sciences [65], has facilitated the collection, quality control, and archiving of large amounts of life science data such as translational medicine data [66].

## Technical challenges related to data analysis and computing infrastructures

Basic as well as clinical researchers need new computational tools to improve data access and aid user-friendly data analysis for efficient decision making in the clinic. Clinicians need new tools that track, trace, and provide fast feedback for individual patient care. Researchers need tools that can be adapted for different data sets and analyses such as those used in a wide range of EU-funded projects through the Innovative Medicines Initiative (IMI)-funded eTRIKS consortium platform [67]. Accessing tool repositories to search for the best tool to answer specific research or clinical questions will be a prerequisite. Equally important are traceable computational environments that maintain data provenance information from patient to sample and from sample to clinically actionable results. In December 2012, the UK announced the 100,000 Genomes Project [68], which aims to sequence 100,000 genomes, from around 70,000 people, with the focus on patients with rare diseases or cancer. The US and China have recently announced plans for similar studies on one million individuals. The goal of these projects is to yield further insights into human health and disease and to build a framework with which to integrate genomics into standard public healthcare programs in the near future. Data continue to increase at an exponential rate and the need for cross-border exchange of biomedical and healthcare data, cloud-storage, and cloud-computing is inevitable [69, 70]. Until many issues of data safety and security are solved, however, local solutions will be favored [71].

## Data quality, acquisition, curation, and visualization

The quality and structure of health data available is inconsistent. A major challenge for preclinical and clinical research is to obtain and achieve access to sufficient high quality, informative data. Owing to a lack of harmonized methods, in most cases health data cannot be directly used for secondary purposes, such as quality of care, pharmacovigilance, safety and efficacy of treatments, health technology assessment, and public health policy. Efforts are underway, in both Europe and the US, to develop and implement standardized data collection, storage, and analysis [10, 72, 73]. The European Open Science Cloud, created by the EC, will offer Europe’s 1.7 million researchers and 70 million science and technology professionals a virtual environment to store, share, and re-use their data across disciplines and borders [74].

Data curation is often neglected but vitally important to warrant high-quality, informative data [75]. Research funders need to make sure that sufficient attention is paid to data quality at the experimental and study design stages, for example, by ensuring data management plans and appropriately reviewed data sharing procedures are in place for all funded research.

**Fig. 1:**
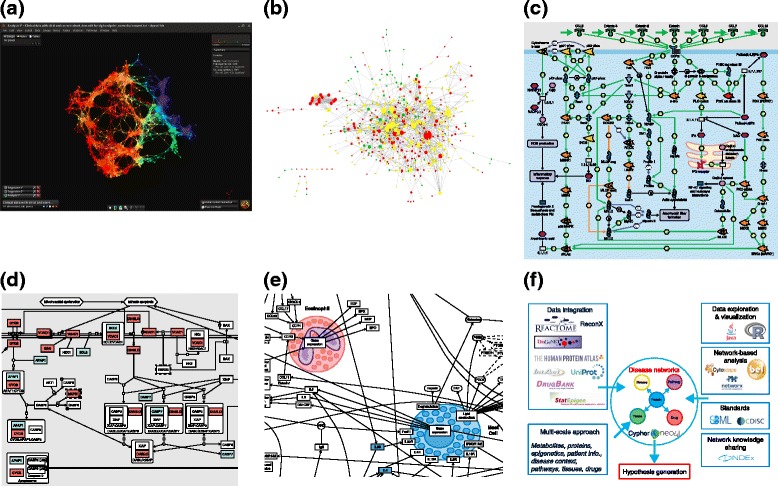
Making sense of complex data and overcoming the hairball syndrome using systems biology algorithms and visualization tools. **a** Visualization of the topology of clinical data from the U-BIOPRED consortium adult severe asthma cohorts (courtesy of Ratko Djukanovic, University of Southampton, UK and Peter Sterk, Amsterdam Medical Center, The Netherlands) [126] using Topology Data Analysis from Ayasdi [127, 128]. **b** Network obtained though integration of genome, transcriptome, and proteome data from the SysCLAD consortium lung transplantation cohorts (courtesy of Johann Pellet, EISBM, France) [129, 130] using Ingenuity® Variant Analysis [131]. **c** Typical static representation of a molecular pathway in Thomson Reuters GeneGo MetaCore™ [132]. **d** An example of a detailed representation of biochemical reactions in the LCSB Parkinson’s molecular map [133]. **e** A cellular-level representation of biological interactions in the EISBM AsthmaMap (courtesy of Alexander Mazein, EISBM, France) [134, 135]. **f** A network representation of data and statements developed as part of a biocentric knowledge base within the eTRIKS consortium (courtesy of Mansoor Saqi and Irina Balaur, EISBM, France) [67]

“*Seeing is believing*”. This phrase is relevant not only for high-resolution microscopy and imaging technologies but also for the presentation and visualization of health-related data. We need to progress from the current display of “hairballs”, incomprehensible comprehensive networks, or ranking tables that nobody has the time or motivation to look at. If we want to provide clinicians with updated, relevant information and clinical decision support systems, the devices have to be user-friendly and intuitive with an interoperable format. The concept of disease-specific maps, with a common computational framework, might be one way to make progress, as demonstrated in several EU-funded projects (Fig. 1).

## Computational modeling and simulation

One of the pathways for exploitation of big data is its combination with predictive, mechanistic models [76] such as those provided by the European Molecular Biology Laboratory–European Bioinformatics Institute (EMBL-EBI) [77]. The Virtual Physiological Human (VPH) community has also endeavored to develop a descriptive, integrative, and predictive computational framework of human anatomy, physiology, and pathology with support from the EC Directorate General for Communications Networks, Content & Technology (DG CONNECT) [78, 79], following the path opened by the IUPS Physiome Project [80]. Predictive computational approaches are associated with infrastructural challenges, particularly for the integration of data with analytical tools and workflows. Online environments such as VPH-Share and projects such as p-medicine have appropriate infrastructures for these applications [81].

Another approach to make sense of big data is based on a systems-level understanding of health and disease [82]. Systems medicine integrative approaches are gradually gaining visibility and enable translation of the human biology complex and voluminous data into a toolbox to demonstrate clinical impact [83]. However, a full appreciation of the power of systems biology and computational modeling for the upcoming changes in health research and healthcare is still missing. Currently, with the exception of oncology, there are still few highly convincing use cases where systems biology approaches have found applications in routine clinical care [45, 46, 84, 85]. Mathematical, computational disease models are unlikely to be routine in health research anytime soon. Achieving necessary changes will need strong support from funders to foster this paradigm shift in methodology.

## Legal and regulatory aspects

A crucial aspect to be addressed concerns the regulatory acceptance of big data for the evaluation of novel pharmacological or biological therapies to complement large randomized clinical trials [86]. Collaborative pilot projects that test the use of big data in observational and/or interventional large clinical trials with the contribution of regulatory agencies can bridge different methodological approaches and determine adapted quality standards. Universities and hospitals do not have the procedures in place to effectively capture and share data with other organizations and countries. We need to develop and adopt high quality standards for data generation and processing to ensure that meaningful and valid data with well-defined semantics are processed and shared. The quality of data generation as well as the processing and regulatory acceptance of big data are addressed at the international level. Research initiatives such as the International Cancer Genome Consortium (ICGC, 2016) [87], the International Human Epigenome Consortium (IHEC, 2016) [88], the Genomic Standards Consortium (GSC, 2016) [89], and the Clinical Data Interchange Standards Consortium (CDISC) [90] and by ISO standards committees (e.g., ISO TC276 WG5, 2016) provide some examples [91]. The recently published FAIR Data Principles of Findability, Accessibility, Interoperability and Reusability for scientific data management should help stakeholders from academia, industry, funding agencies, and non-commercial publishers support the reuse of scholarly data [92]. Given the complexity and high number of stakeholders involved in the implementation of data standards within hospital and university settings, the biggest chance for success comes with highly focused pilot projects. Key factors include flexibility, expansion through modular strategies, and the identification and involvement of key healthcare actors providing them with immediate benefits.

Linking existing initiatives and building new initiatives on clinical data interchange are also important. The Global Alliance for Genomics and Health (GA4GH, 2016) [93] initiative is working towards technical, ethical, and legal frameworks to address and resolve some of these issues. The Coordinated Research Infrastructures Building Enduring Life-science (CORBEL) Services, a recently launched European consortium, will also contribute to the above data-sharing challenges [94]. CORBEL is an initiative of 11 new Biological and Medical Science Research Infrastructures (BMS RIs), who together will create a platform for harmonized user access to biological and medical technologies, biological samples, and data services (e.g., BRIDGEHEALTH consortium [95]). The Genomics England policy [68] of storing all data within the National Health Service (NHS) with highly regulated restricted access to prevent abuse of private information and user protocols might be a way to go forward. This policy will need to be complemented by that of the UK Personal Genome Project allowing volunteers to donate their personal genome from Genomics England and other sources to the public domain [96].

The processes and legal agreements for data sharing across registries and European Member States are seldom established. The harmonization of regulatory frameworks is crucial while also ensuring personal data protection and compliance with current legal frameworks, which includes provisions on how to prevent, handle, and prosecute potential abuse of the system. For example, there is no consensus within international law on whether specific requirements should be applicable to genetic information. Several documents exist at the regional and international levels that include useful guidelines, such as the UNESCO International Declaration on Human Genetic Data (2003) [97] and the Organisation for Economic Co-operation and Development (OECD) Guidelines on Human Biobanks and Genetic Research Databases (2009) [98]. The GA4GH has developed the Framework for Responsible Sharing of Genomic and Health-Related Data [99].

## Privacy protection and data sharing policies

There are broad differences within and across Europe with regards to privacy protection and data sharing polices [100]. The workshop in Luxembourg emphasized that the “one size fits all” approach will not be applicable in Europe. The EC proposal for the General Data Protection Regulation (2012/0011COD) [101] attempts to harmonize the fragmented situation that exists under the current Data Protection Directive (95/46/EC, European Parliament and Council, 1995). In the compromise text concluded in the trilogue negotiations between Parliament and the Council, a paragraph is included in the preamble of the new act which defines DNA and RNA as personal data [102]. A Code of Practice on Secondary Use of Medical Data in European Scientific Research Projects has been developed [103] and is being deployed in the IMI-funded project eTRIKS [67]. There is also a need to have a much higher level of security than is possible today. One suggestion was to explore block-chain technology, which makes use of a digital, distributed transaction record, digital events, with identical copies maintained on multiple computer systems, shared between many different parties. Once entered, the block-chain contains a certain and verifiable record of every single transaction [104]. Originally used as the technology underlying “Bitcoin”, the potential to make secure transactions of biomedical and healthcare data is being explored [105]. Another possibility would be to make use of differentiated privacy approaches as practiced in health information exchanges [106].

## Research infrastructures

Similarly, research infrastructures are instrumental to support the harmonization of legal and ethical frameworks in European countries, as demonstrated by the Common Service on Ethical, Legal and Social Implications (CS ELSI) of Biobanking and BioMolecular resources Research Infrastructure Consortium (CS ELSI BBMRI-ERIC, 2016) [107]. The goal of ELSI BBMRI-ERIC is to facilitate and support cross-border exchanges of human biological resources and data attached for research uses, collaborations, and sharing of knowledge, experiences, and best practices.

Existing computational infrastructures are coping with storage of big data, but the challenge within the EU is the lack of a large-scale European infrastructure and methods of secure data distribution in a cross-border setting [108]. It is crucial to ensure that the infrastructures that exist and evolve are coordinated and sustainable. Initiatives such as ELIXIR [65] and the CS IT BBMRI-ERIC [109] have begun to address these issues but there is a need for coordination and significant strategic investments to ensure that organizations such as these are equipped to support the rapid growth and evolution of healthcare informatics over the next decade. Distribution of expertise and facilities, consistent operation, and federation throughout Europe are essential for scalability and long-term sustainability. This has become one of the key challenges of distributed infrastructures such as BBMRI-ERIC [110], ECRIN [111], and ELIXIR [65], which could benefit from the long experience of CERN in particle physics as discussed earlier [52].

## Training and education: many health data, insufficient health data scientists

One of the biggest bottlenecks and challenges is the availability of healthcare professionals and clinical researchers that are able to use the latest information technologies developed in the big data analytics era [112, 113]. Data managers with good insight into the specificities of the health application domain are rare. An equally important bottleneck will be the lack of trained clinical scientists to deal with big data. The majority of university hospitals face a daily struggle to balance their budget. Clinical research rarely brings in money to pay the costs for clinical care. As a result, many university hospitals cease to maintain their culture of research as an essential basis for top-level healthcare. Once the chain of training the next generation of clinical scientists is broken by the retirement of the current trainers, the situation will change dramatically and result in a catastrophic shift. Therefore, there is a pressing need for programs that support the careers of clinical scientists with state-of-the-art training in data analysis and management.

There is a clear lack of cross-disciplinary education and training, which means that employees in the clinical environment often do not have the expertise to deal with big data in clinical research and healthcare. Coordinating Action Systems Medicine (CASyM) [83] has developed modules of multidisciplinary training for the next generation of researchers and medical doctors. Furthermore, despite compulsory requirements of data transparency applicable to clinical trials data, researchers and clinicians often have little incentive to make data fully available. Another challenge may be public skepticism about the security of an integrated healthcare system. However, several global initiatives have shown that individuals are ready to share their medical data for advancing science (Personal Genome Project) [96], which highlights the potential contribution of citizen science to big data in health research. Data donor cards would provide an incentive for people to make their data publicly available and would work in the same way as organ donor cards, thereby reusing a system already understood by many people. Legislative approaches should include opt-in and or opt-out solutions. For a successful transformation of healthcare, we need to push the boundaries of interdisciplinarity, which comprises the natural sciences such as biology and medicine, engineering, the social sciences, and the humanities. Projects fail more often because of the underappreciation of the complexities of ethical, legal, and social factors than for technological reasons.

The workshop in Luxembourg brought together a wide range of experts and stakeholders to discuss the key developments, challenges, and potential solutions that we face with using big data for the benefit of the patients, the health care industry, and Europe as a whole. The workshop resulted in specific recommendations for European policy-makers. There was no doubt among the participants that big data and the revolution in ICT will transform healthcare. There was also a sense of urgency to implement rapidly the possible and to tackle the yet impossible.

## Recommendations for an EU action plan

### Launch pilot projects on the application of big data to inform health

The primary recommendation is for the launch of pilot projects on the application of big data that involve healthcare providers, health technology developers, policy-makers, and advisory bodies. Pilot translational research projects that involve healthcare workers and patients could bring big data closer to the clinic and prove the value of collecting and analyzing such information using the latest mathematical and computational tools. The design principles for achieving integrated healthcare information systems [114] might serve as guidance on how small pilot projects can be used for future expansion.

### Leverage the potential of open and citizen science for the exploitation of big data in health

The concept of “open science” includes open access to publications and raw data, transparency of tools and methodologies, and networking of researchers across fields and countries [115]. Open science provides significant added value in pilot studies and its broad implementation in the scientific community and society is under discussion. For example, a high-profile effort to switch all peer-reviewed publishing to open access within the next years is envisaged [116–119].

The second recommendation is to encourage leveraging the complementarity between open and citizen science in the context of big data in health. It will be important to inform and involve the public not only about data collection but also about all aspects of health research [120]. Consumer genomics companies are already successful at gathering metadata through engagement with their customers. The field of rare diseases has also benefitted greatly from the involvement of parents of children with such diseases, using non-traditional techniques such as social media to build a network of related cases of a particular syndrome. “Citizen science” is also becoming increasingly important because of the increased uptake of mobile health devices, consumer electronics, and household appliances and is well-aligned with the EC focus on “responsible research and innovation” that includes elements of open science in its ongoing Horizon 2020 Framework Programme policy [121].

### Catalyze the involvement of all relevant stakeholders in projects

The third recommendation is to involve in projects all relevant stakeholders, which includes clinicians, patient organizations, researchers, software providers, healthcare managers, ethical and legal experts, regulatory authorities, policy-makers, pharmaceutical companies, and funding bodies. Multidisciplinary involvement is required to secure an effective translation from basic research to applied healthcare and to bridge the organizational and cultural differences in data sharing practices across Europe and within the different health sectors in a worldwide context. Clark and colleagues have laid out “*a core set of lessons that should become part of a basic training for researchers interested in crafting usable knowledge for sustainable development*” [122]*.* One of the most important lessons is to understand that research is a social and political process, not just a process of discovery, and that stakeholders are diverse and need to be involved in the team building process at an early stage.

It is likely that bioinformaticians, biostatisticians, and computational scientists will more often be included in the near future as natural members of research and clinical teams and healthcare administration, as already carried out by global pharmaceutical organizations. Important towards this direction is cross-disciplinary training and to improve the dialogue between the information technology experts, biologists, and clinicians, especially as these groups have the potential to affect greatly the practical outcome of research.

### Support a rapid transition to new computational, statistical, and other mathematical methods of analysis

The fourth recommendation is to foster the transition to new computational, statistical, and other mathematical methods of analysis that enable the integration of data across the multiple scales of time and space typical of complex biological systems in their healthy and diseased states [123]: traditional methods of analysis are no longer scalable for such big data diversity. The roadmap developed by the Avicenna Coordination Support Action provides a vision on how computer simulation will transform the biomedical industry by developing “*in silico* clinical trials” [124].

The need for new methods spans a wide range of topics. We need effective methods for data integration, collection, and data provenance management, for example, the integration of genomics information and patient registries with EHRs and the integration of model organism data into disease models. We also need improved methodologies and tools to support data entry by those recording data, such as visual and physiological information. Innovative statistical methods, such as models for predictive analytics and computational models tailored to big data, are required to enable hypothesis generation, estimation of risk models, and study design. The Infrastructure, Design, Engineering, Architecture, and Integration project (IDeAl) [125] is taking steps in this direction by developing new methods for gene selection to tailor the design for small population group trials. There may even be a requirement for new types of data and data formats. The development and use of interoperable data, technology standards, and harmonized operating procedures for data collection and analysis are paramount to enable data integration and to support data flow and federated access between public and private partners. Furthermore, applicable data protection standards and maintaining public trust are important to realize the full potential of big data in health research for European citizens and, by extension, worldwide. In this regard, we need a definition of core data sets that could serve as a common standard for any individual health state.

Using the big data revolution to drive the transformation of healthcare requires resources for state-of-the-art ICT infrastructure, training programs, and pilot projects that can serve as a role model. These costs, however, will be overcompensated by the gains that will come with the implementation of functioning digital workflows and sophisticated health data analytics and the creation of a new health and wellness industry.

### Accelerate the harmonization of regulatory frameworks in Europe for health-related research and data sharing

The final recommendation is to agree on the necessity for, and the high priority of, accelerating the harmonization of the European policy and regulatory frameworks that affect health-related research and data sharing and the distribution of biological material used for the generation of data necessary for research. There should be a balance between the protection of an individual’s privacy, while acknowledging that many patients are much more open about data sharing than current policies seem to assume, and the ability to proceed with research to ensure that Europe remains competitive in health research. EU and national funding bodies should take stock of the existing best practices and catalyze their adoption in transnational health research.

## Conclusions and future perspectives

The digital revolution is underway. A number of industries have already transformed their activities or have now become inoperative. The driving forces are miniaturization, automation, and now increasingly the convergence of artificial intelligence, deep learning, and robotics. Healthcare will not escape these developments. In fact, big data as a driving force will play an even more important role than in most industries. In Europe, working across borders is the only way to master the challenges of this scientific, technological, and industrial revolution. The single most important factor is the workforce. Countries that are ahead in ICT competence and have an understanding of cultural differences and an ability and willingness to work together have the best chance to succeed.

## Abbreviations

BBMRI, Biobanking and Biomolecular Resources Research Infrastructure; BMS RI, Biological and Medical Sciences Research Infrastructure; CASyM, Coordinating Action Systems Medicine; CDISC, Clinical Data Interchange Standards Consortium; CORBEL, Coordinated Research Infrastructures Building Enduring Life-science Services; EBI, European Bioinformatics Institute; EC, European Commission; EHR, electronic health record; EISBM, European Institute for Systems Biology and Medicine; ELSI, ethical, legal, and social implications; EMBL, European Molecular Biology Laboratory; ENCR, European Network of Cancer Registries; EORTC, European Organisation for Research and Treatment of Cancer; ERIC, European Research Infrastructure Consortium; EU, European Union; EURORDIS, Rare Diseases Europe; GA4GH, Global Alliance for Genomics and Health; ICGC, International Cancer Genome Consortium; IHEC, International Human Epigenome Consortium; IMI, Innovative Medicines Initiative; ISO, International Organization for Standardization; LCSB, Luxembourg Centre for Systems Biomedicine; NHS, National Health Service; OECD, Organization for Economic Co-operation and Development; UNESCO, United Nations Educational, Scientific and Cultural Organization; VPH, virtual physiological human
