# Erratum to: Making sense of big data in health research: towards an EU action plan

**DOI:** 10.1186/s13073-016-0376-y

**Published:** 2016-11-07

**Authors:** Charles Auffray, Rudi Balling, Inês Barroso, László Bencze, Mikael Benson, Jay Bergeron, Enrique Bernal-Delgado, Niklas Blomberg, Christoph Bock, Ana Conesa, Susanna Del Signore, Christophe Delogne, Peter Devilee, Alberto Di Meglio, Marinus Eijkemans, Paul Flicek, Norbert Graf, Vera Grimm, Henk-Jan Guchelaar, Yi-Ke Guo, Ivo Glynne Gut, Allan Hanbury, Shahid Hanif, Ralf-Dieter Hilgers, Ángel Honrado, D. Rod Hose, Jeanine Houwing-Duistermaat, Tim Hubbard, Sophie Helen Janacek, Haralampos Karanikas, Tim Kievits, Manfred Kohler, Andreas Kremer, Jerry Lanfear, Thomas Lengauer, Edith Maes, Theo Meert, Werner Müller, Dörthe Nickel, Peter Oledzki, Bertrand Pedersen, Milan Petkovic, Konstantinos Pliakos, Magnus Rattray, Josep Redón i Màs, Reinhard Schneider, Thierry Sengstag, Xavier Serra-Picamal, Wouter Spek, Lea A. I. Vaas, Okker van Batenburg, Marc Vandelaer, Peter Varnai, Pablo Villoslada, Juan Antonio Vizcaíno, John Peter Mary Wubbe, Gianluigi Zanetti

**Affiliations:** 1 European Institute for Systems Biology and Medicine, 1 avenue Claude Vellefaux, 75010 Paris, France; 2 CIRI-UMR5308, CNRS-ENS-INSERM-UCBL, Université de Lyon, 50 avenue Tony Garnier, 69007 Lyon, France; 3 Luxembourg Centre for Systems Biomedicine, University of Luxembourg, 7 Avenue des Hauts Fourneaux, 4362 Esch-sur-Alzette, Luxembourg; 4 Wellcome Trust Sanger Institute, Wellcome Genome Campus, Hinxton, Cambridge, CB10 1SA UK; 5 Health Services Management Training Centre, Faculty of Health and Public Services, Semmelweis University, Kútvölgyi út 2, 1125 Budapest, Hungary; 6 Centre for Personalised Medicine, Linköping University, 581 85 Linköping, Sweden; 7 Translational & Bioinformatics, Pfizer Inc., 300 Technology Square, Cambridge, MA 02139 USA; 8 Institute for Health Sciences, IACS - IIS Aragon, San Juan Bosco 13, 50009 Zaragoza, Spain; 9 ELIXIR, Wellcome Genome Campus, Hinxton, Cambridge, CB10 1SD UK; 10 CeMM Research Center for Molecular Medicine of the Austrian Academy of Sciences, Lazarettgasse 14, AKH BT25.2, 1090 Vienna, Austria; 11 Department of Laboratory Medicine, Medical University of Vienna, Lazarettgasse 14, AKH BT25.2, 1090 Vienna, Austria; 12 Max Planck Institute for Informatics, Campus E1 4, 66123 Saarbrücken, Germany; 13 Príncipe Felipe Research Center, C/Eduardo Primo Yúfera 3, 46012 Valencia, Spain; 14 University of Florida, Institute of Food and Agricultural Sciences (IFAS), 2033 Mowry Road, Gainesville, FL 32610 USA; 15 Bluecompanion Ltd, 6 London Street (second floor), London, W2 1HR UK; 16 Technology, Data & Analytics, KPMG Luxembourg, Société Coopérative, 39 Avenue John F. Kennedy, 1855 Luxembourg, Luxembourg; 17 Department of Human Genetics, Department of Pathology, Leiden University Medical Centre, Einthovenweg 20, 2333 ZC Leiden, The Netherlands; 18 Information Technology Department, European Organization for Nuclear Research (CERN), 385 Route de Meyrin, 1211 Geneva 23, Switzerland; 19 Julius Center for Health Sciences and Primary Care, University Medical Center Utrecht, Heidelberglaan 100, 3508 GA Utrecht, The Netherlands; 20 European Molecular Biology Laboratory, European Bioinformatics Institute (EMBL-EBI), Wellcome Genome Campus, Hinxton, Cambridge, CB10 1SD UK; 21 Department of Pediatric Oncology/Hematology, Saarland University, Campus Homburg, Building 9, 66421 Homburg, Germany; 22 Project Management Jülich, Forschungszentrum Jülich GmbH, Wilhelm-Johnen-Straße, 52428 Jülich, Germany; 23 Department of Clinical Pharmacy & Toxicology, Leiden University Medical Center, Albinusdreef 2, 2333 ZA Leiden, The Netherlands; 24 Data Science Institute, Imperial College London, South Kensington, London, SW7 2AZ UK; 25 CNAG-CRG, Center for Genomic Regulation, Barcelona Institute for Science and Technology (BIST), C/Baldiri Reixac 4, 08029 Barcelona, Spain; 26 Institute of Software Technology and Interactive Systems, TU Wien, Favoritenstrasse 9-11/188, 1040 Vienna, Austria; 27 The Association of the British Pharmaceutical Industry, 7th Floor, Southside, 105 Victoria Street, London, SW1E 6QT UK; 28 Department of Medical Statistics, RWTH-Aachen University, Universitätsklinikum Aachen, Pauwelsstraße 30, 52074 Aachen, Germany; 29 SYNAPSE Research Management Partners, Diputació 237, Àtic 3ª, 08007 Barcelona, Spain; 30 Department of Infection, Immunity and Cardiovascular Disease and Insigneo Institute for In-Silico Medicine, Medical School, University of Sheffield, Beech Hill Road, Sheffield, S10 2RX UK; 31 Department of Statistics, School of Mathematics, University of Leeds, Leeds, LS2 9JT UK; 32 Department of Medical & Molecular Genetics, King’s College London, London, SE1 9RT UK; 33 Genomics England, London, EC1M 6BQ UK; 34 National and Kapodistrian University of Athens, Medical School, Xristou Lada 6, 10561 Athens, Greece; 35 Vitromics Healthcare Holding B.V., Onderwijsboulevard 225, 5223 DE ’s-Hertogenbosch, The Netherlands; 36 Fraunhofer Institute for Molecular Biology and Applied Ecology ScreeningPort, Schnackenburgallee 114, 22525 Hamburg, Germany; 37 ITTM S.A., 9 avenue des Hauts Fourneaux, 4362 Esch-sur-Alzette, Luxembourg; 38 Research Business Technology, Pfizer Ltd, GP4 Building, Granta Park, Cambridge, CB21 6GP UK; 39 Health Economics & Outcomes Research, Deloitte Belgium, Berkenlaan 8A, 1831 Diegem, Belgium; 40 Janssen Pharmaceutica N.V., R&D G3O, Turnhoutseweg 30, 2340 Beerse, Belgium; 41 Faculty of Life Sciences, University of Manchester, AV Hill Building, Oxford Road, Manchester, M13 9PT UK; 42 UMR3664 IC/CNRS, Institut Curie, Section Recherche, Pavillon Pasteur, 26 rue d’Ulm, 75248 Paris cedex 05, France; 43 Linguamatics Ltd, 324 Cambridge Science Park Milton Rd, Cambridge, CB4 0WG UK; 44 PwC Luxembourg, 2 rue Gerhard Mercator, 2182 Luxembourg, Luxembourg; 45 Philips, HighTechCampus 36, 5656AE Eindhoven, The Netherlands; 46 Department of Public Health and Primary Care, KU Leuven Kulak, Etienne Sabbelaan 53, 8500 Kortrijk, Belgium; 47 INCLIVA Health Research Institute, University of Valencia, CIBERobn ISCIII, Avenida Menéndez Pelayo 4 accesorio, 46010 Valencia, Spain; 48 Swiss Institute of Bioinformatics (SIB) and University of Basel, Klingelbergstrasse 50/ 70, 4056 Basel, Switzerland; 49 Agency for Health Quality and Assessment of Catalonia (AQuAS), Carrer de Roc Boronat 81-95, 08005 Barcelona, Spain; 50 EuroBioForum Foundation, Chrysantstraat 10, 3135 HG Vlaardingen, The Netherlands; 51 Integrated BioBank of Luxembourg, 6 rue Nicolas-Ernest Barblé, 1210 Luxembourg, Luxembourg; 52 Technopolis Group, 3 Pavilion Buildings, Brighton, BN1 1EE UK; 53 Hospital Clinic of Barcelona, Institute d’Investigacions Biomediques August Pi Sunyer (IDIBAPS), Rosello 149, 08036 Barcelona, Spain; 54 European Platform for Patients’ Organisations, Science and Industry (Epposi), De Meeûs Square 38-40, 1000 Brussels, Belgium; 55 CRS4, Ed.1 POLARIS, 09129 Pula, Italy; 56 BBMRI-ERIC, Neue Stiftingtalstrasse 2/B/6, 8010 Graz, Austria

## Erratum

The published article [1] has two points of confusion in the section entitled “Technical challenges related to the management of electronic health records”. Firstly, the International Rare Diseases Research Consortium (IRDiRC) has developed policies and guidelines on approaches to data sharing meant to enable and improve the development of diagnoses and therapies for rare diseases. However, at present, IRDiRC has not developed best practices for the management of electronic health records (EHRs). Secondly, RARE-Bestpractices is a European Commission 7th Framework Programme (FP7) funded initiative, independent of IRDiRC. RARE-Bestpractices contributes to IRDiRC goals and objectives; however the initiative itself is not sponsored nor connected to IRDiRC.
